# Opening the gender diversity black box: causality of perceived gender equity and locus of control and mediation of work engagement in employee well-being

**DOI:** 10.3389/fpsyg.2015.01371

**Published:** 2015-10-09

**Authors:** Radha R. Sharma, Neha P. Sharma

**Affiliations:** ^1^Raman Munjal (Hero MotoCorp) Chair Professor and Professor, Organizational Behavior and HRD, Management Development InstituteGurgaon, India; ^2^Research Scholar, Management Development InstituteGurgaon, India

**Keywords:** gender equity, employee well-being, work engagement, optimism, locus of control, gender diversity, talent management, Indian study

## Abstract

The study is aimed at assessing the role of perceived gender equity and locus of control in employee well-being at the workplace and ascertaining if work engagement mediates between perceived gender equity, locus of control, and employee well-being (measured through optimism, general satisfaction with life and work, and executive burnout). Adopting a personal survey method data was collected from 373 managers (both males and females) from the public and private sectors representing manufacturing and service industry in India. The study bridges the knowledge gap by operationalizing the construct of perceived gender equity and studying its role in the work engagement and employee well-being. Conceptualization of the well-being in an unconventional way covering both the positive and the negative aspects extends the understanding of the emerging concept of well-being. It has practical implications for talent management and work engagement besides promoting gender equity at the workplace for employee well-being. It opens vistas for the gender based theory and cross cultural research on gender equity.

Diversity at workplaces in India exists in various forms namely the cultural, the linguistic^1^, the religious, and the gender diversity ^2^ as Indian Constitution's Article 15, 16, and 39 grant equality to all in employment. Though gender diversity is an integral part of workplaces, women in India have not been able to optimally utilize the fundamental right conferred upon them due to several socio-economic obstacles, prejudices and covert discrimination (Sharma Radha, 1982). India ranked 113th out of 135 countries covered in the World Economic Forum's Global Gender Gap Index, 2011 (Hausmann et al., 2011). In the Global Gender Gap Report (Hausmann et al., 2012) of the World Economic Forum, positioning countries on the basis of their capacity for bridging the gender gap on four key parameters viz., (i) accessibility to healthcare; (ii) education; (iii) economic equality, and (iv) political participation, India ranked 105th, which is the lowest amongst the BRIC nations (Cann, 2012; Hausmann et al., 2012). Research evidence suggests that Women's career progress is more complex than that of their counterpart because of the barriers they face (Broadbridge and Fielden, 2015).

A study of Indian IT professionals by the Dataquest-Jobs Ahead (2003) found that of 150,000 employees, women constituted over 19% of the total workforce at the lower levels (up to 3 years of experience). Their number dropped to 6% at the senior level (with more than 10 years of experience). According to Som Mittal (2008) the President, National Association of Software and Services Companies (NASSCOM) out of two million employed by the business process outsourcing (BPO) industry in India about half of the employees are women. “However, at the leadership level women representation is only about 6% and we want to focus on this aspect.” Tata Consultancy Services (2010) conducted a survey commissioned by People Matters on “Benchmarking Gender Inclusion in the Corporate India” in 116 companies including the largest employers and found under- representation of women in senior positions and at the board level. Therefore, women organizations create an environment to attract, retain and groom women in the workplace. Gender equity is not a matter of corporate social responsibility alone but it makes a good business sense. McKinsey and Company (2012) in an Indian study of top 30 firms on the Bombay Stock Exchange found that the family owned firms with women leaders fared consistently better than other firms for a 5 consecutive years. Another rationale for employing women is to gain competitive edge. Indian as well as multinational companies operating in India have been focusing on recruitment, development and retention of talent which is in short supply (Society for Human Resource Management (SHRM), 2009) despite no labor shortage in India. Thus, it is imperative for companies to engage in key component of economic growth- the talent of women (Bagati and Carter, 2010). India's market regulator, the Securities and Exchange Board of India (SEBI), had set a deadline of April 1, 2015 for all the 9000 country's listed companies to appoint at least one female director. According to media reports (Press Trust of India, 2015) 832 women were appointed to 912 directorship positions in 872 companies by the due date. Due to shortage of female talent, women with long experience in areas such as banking and finance are serving on the boards of several companies which makes a case for gender equity at the senior level in Indian companies.

Also, of the total enrolment in higher education 40.3% are women (Govt. of India, 2009-2010) and an increasing percentage of women are opting for MBA programme (from 16 to 19% during 2004-2006); these figures are rising every year indicating that women are aspiring for leadership roles in organizations. But the data shows that they are stuck at the junior and middle levels (Bagati and Carter, 2010) and a compelling factor for gender equity is that Indian women are leaving their career much sooner than professional women in other Asian countries (Francesco, 2011). This underscores the importance of gender equity at the workplace in India as the share of women in non-agricultural employment is only 19% (World Bank, 2010). As per Census of India (2011a,b,c) the work participation rate for women is 25.56 as against 51.7 for males which reveals that the women work participation is about half of that of the male. Also, due to the workplace inequity many of them quit their job; this calls for empirical research to identify the causes so that measures could be taken to mitigate it.

International researches reveal positive effect of the work participation of women on business performance. Through a longitudinal study of 25 Fortune 500 firms, Adler (2009) at Pepperdine University showed a strong correlation between women on the executive board and profitability in both the short and the long term indicating that gender equity is driven by the business needs of a company. Research reveals that diversity (gender and racial) leads to a competitive edge in terms of sales revenues, number of customers and the market shares (Herring, 2009). Thus, the gender equity at the workplace would leverage female talent from an employee pool to address the shortage of talent in the highly competitive business environment. Besides, the gender equity initiatives can help build the corporate brand and improve organizational reputation. Landau (1995) examined the relationship of gender to managers' ratings of promotion potential for a sample of 1268 managerial and professional employees and found that the gender is significantly related to promotion potential as the women are rated lower than the males. The so called, “glass ceiling” (Cotter et al., 2001) has always prevented women from realizing their true potential in the business organizations. Drawing attention toward the disparities in pay; access to education; lack of training opportunities; career breaks and restricted occupational options for the working women, Walby (2003) has emphasized the need for innovative policies for ensuring gender equity at the workplace. Researches in the leisure and sport management sectors also suggest that the gender equity can be ensured only if the organizations address both the “structural” as well as the “cultural” (in) equities prevalent in their systems (Aitchison, 2000). The gender diversity would reduce employee attrition and would attract the talent leading to improved decision-making and innovations. There is an urgent need to understand the phenomenon of the gender equity at workplace and its role in individual and organizational outcomes.

The foregoing literature review and the absence of scholarly work on gender equity at the workplace in India make a compelling business case for a study on gender equity. This has provided impetus to undertake this research to study if gender equity would enhance work engagement, satisfaction with life/work, optimism, employee well-being and would prevent executive burnout. The findings of the study are expected to reduce employee absence and the medical expenses. As the subject of gender equity is sensitive it was decided to adopt personal survey method for data collection in face to face situation so that quality data could be obtained with maximum response rate.

A perusal of gender researches across the globe reveals that most studies have focused on the attitude toward women (Spence and Hahn, 1997), work–family conflict, commitment and supervisory support. Fiksenbaum et al. (2010) studied relation between the two virtues of optimism and proactive behavior and psychological well-being amongst working women in the Turkish banking sector. Though a few researches focused on pay equity and the gender discrimination, the authors have not come across any causality study on the role of perceived gender equity on work engagement and employee well-being. The research yields that people with the internal locus of control have more positive self-evaluation and better performance (Spector, 1982) and the interaction of the authors with the HR professionals supported that despite glass ceiling and obstacles some women with internal locus of control have made their way up. In view of the importance of the perceived gender equity and the locus of control in performance (Harvey and Thomas, 2004), the present study includes these as independent variables. Thus, the research focuses on causality of the perceived gender equity and locus of control as independent variables with a view to studying their role in the employee well-being, as dependent variable measured by optimism, general satisfaction with life and work, and executive burnout among Indian managers. The study envisages the work engagement as a mediating variable between independent and dependent variables.

## Review of researches and hypotheses development

### Gender equity: the construct and the context

The terms gender equity and gender equality are often used interchangeably but there is a difference. “Gender equity means fairness of treatment for women and men, according to their respective needs. This may include equal treatment or treatment that is different but which is considered equivalent in terms of rights, benefits, obligations and opportunities” (UNESCO, 2000). According to World Health Organization (2001) gender equity is defined as “fairness and justice in the distribution of benefits and responsibilities between women and men.” Figart and Mutari (2000) have mapped gender equity at work through the level of acceptance of “gendered work time practices” in organizations encompassing “total hours worked” and “flexible work options” available to the female and male employees in multinational organizations. Similarly, Baker et al. (2002) have tried to capture the construct through an analysis of the number of women at upper echelon and salary drawn by them in federal labor relations in the United States.

Gender equality is defined as “a human right” according to United Nations. Therefore, one expects equal pay for equal work, right to vote and dignified treatment of women, but one finds that women are the victims of poverty, violence and unemployment in many countries. Debate about gender equity and gender equality has recently moved to gender mainstreaming. UNDP (2013) defines gender mainstreaming as “taking account of gender equality concerns in all the policy, programs, administrative and financial activities, and in organizational procedures, thereby contributing to organizational transformation.”

The Hausmann et al. (2011) covering the world's largest employers in 20 countries reported that India had a meager 23% of women employees as compared to the United States (52%), Spain (48%), Canada (46%), Finland (44%), Austria (29%), and Japan (24%). The women employees have concentration at the entry or middle level positions and remain scarce at the senior management or the board positions in most countries. Thus, in order to mainstream women it is important to know the dimensions of inequity (Zahidi and Herminia, 2010). Similar sentiments have been voiced in the United Nations Development Programme (UNDP, 2010) sponsored Asia-Pacific Human Development Report (2010) which anticipated that the lack of women's involvement in the labor force costs the Asia-Pacific region billions of dollars on an annual basis. Through an analysis of managerial and professional occupations in the United States, Queneau (2008) found that certain occupations are gender-integrated or gender-dominated; occupations like engineering are generally male-dominated while teaching and counseling, female-dominated. Powell et al. (2009) posited though more women have started entering male-dominated professions like engineering, by and large, such occupations are still considered unsuitable for women.

A study of workforce diversity in the Indian context has found empirical support for discrimination on the basis of employees' gender and social class (Kundu, 2003). Stereotyping, attributed social functions, masculine culture, concealed discrimination and power distance are majorly responsible for hindering the path to success for women in India. Indian women are found in typical stereotyped professions like teaching, nursing, personal service and social work and fewer women are found in the higher echelons of science, technology and management (Sharma Radha, 1984; Sharma Radha and Mukherjee, 2012). A study exploring the representation of women in two Indian accounting professional bodies revealed that these had either weak or non-existent representation of women on their websites (Kyriacou et al., 2010). With a view to identifying the challenges and opportunities for Indian women managers, Gupta et al. (1998) surveyed 162 managers (37% female and 63% male) across service and manufacturing organizations. The study concluded that to create a healthy learning organization, perceptual differences regarding the gender issues among managers needed assertive action. In the virtual work space, the gender has been considered an important variable for the employees' adjustment to the virtual work (Raghuram et al., 2000), thereby highlighting the importance of gender diversity across occupational domains. Basu (2008) posits that a research probing into gender perceptions at the workplace will be significant in explaining why gender inequity exists and how such perceptions could be altered. A perusal of the researches reported above indicates that studies so far have focused on stereotypes and low representation of women in the leadership roles but no study so far has empirically investigated the causes of gender inequity at workplaces in India. Sharma Radha (2013) evolved the construct of perceived gender equity at the workplace in India through an empirical research and developed and standardized a measure for it. Based on her research the following definition of the perceived gender equity has been adopted for the present study:

“*Perceived Gender Equity at the workplace is employees' positive perception of equal opportunity in recruitment, training and development, compensation, career progression, dignified treatment and professional respect through the organizational policies, practices and environment” (Sharma Radha*, *2013**).*

### Locus of control, gender and job satisfaction

Locus on control explains whether employees have personal belief that their outcomes are controlled internally or externally. Those with the internal control feel that their outcomes can be controlled by their own ability, skills or efforts; whereas those with the external control feel that their outcomes are beyond their control and external forces control them (Mitchell et al., 1975). The locus of control has been defined as “generalized expectancy for internal as opposed to external control of reinforcements” (Lefcourt, 1976). The characteristics of locus of control (LOC) identified by Rotter (1966) have been high achievement orientation and low outer-directedness. According to Leone and Burns (2000) the locus of control, as a construct, measures the degree to which individuals believe they are responsible for the consequences of their behavior. People with the internal locus of control have more positive self-evaluation, better performance, are more satisfied with their job and have better coping skills in stressful situations (Spector, 1982) and tend not to burnout (Glassgow, 1986). In a study of gender differences Majzub et al. (2011) found that the males score higher on both internal and external (LOC) than the females. Overall, this study substantiated earlier research findings of positive relationship between LOC and academic achievement. Locus of control has been extensively studied in relation to health (Georgio and Bradley, 1992); hypertension (Stantion, 1987); physical and mental health and quality of life in persons suffering from HIV, kidney disease, epilepsy migraine and diabetes (Maltby et al., 2007). Judge and Bono (2001) found a positive correlation (0.32) between the internal LOC and job satisfaction relating only to work. The foregoing studies have largely focused on the LOC and physical diseases except one which has studied its relationship with job satisfaction. This shows the research gap as “satisfaction with life and work” as an integrated construct and a measure of “well-being” at the workplace has not been studied in relation to internal locus of control. In view of the foregoing gender differences and the knowledge gap, locus of control has been chosen as the second independent variable, and well-being (satisfaction with life and work) as a dependent variable.

Whether the perception of equity affects the work engagement and whether the work engagement mediates between the perceived gender equity and the well–being are under-researched areas, hence the work engagement and employee well-being have been included in the present study.

### Work engagement and gender

Schaufeli et al. (2002) define the work engagement as the “positive, fulfilling, work-related state of mind that is characterized by vigor, dedication and absorption.” Therefore, engaged employees are enthusiastic about their work and exhibit high levels of energy. Later Schaufeli et al. (2006) have empirically evolved the construct of work engagement and have developed a scale to measure the same. Discrimination Alert (2008) has reported gender differences in employee engagement in Australia. Though there are studies on the employee engagement in general but there is knowledge gap linking the perceived gender equity, work engagement and employee well-being.

### Well-being

Well-being is an emerging construct but there is no universally accepted definition of it so far. It has commonly been described as a positive and sustainable condition which equips individuals and groups to thrive even in the face of adversity. There are multiple perspectives provided by the scholars from various fields. The “Subjective well-being” in Positive Psychology deals with understanding, explaining and predicting subjective well-being and facilitating happiness rather than rectifying deficits (Seligman, 2002; Gable and Haidt, 2005; Seligman et al., 2005; Positive Psychology Centre Report, 2009). Ryff (1995) has conceptualized psychological well-being as a multi-dimensional model involving self-acceptance, personal growth, purpose in life, positive relations, environmental mastery and autonomy. Ryff and Keyes (1995) and Ryff and Singer (1996) have defined it as “a generalized feeling of happiness” (Schmutte and Ryff, 1997). The well-being has also been referred to as the life satisfaction, the cognitive evaluation of one's overall life (Diener et al., 1985) and the self-acceptance (Ruderman et al., 2002) which include aspects like openness to new experiences, autonomy, initiative and positive relationships. Sharma Radha (2011) has conceptualized mental well-being as the multi-dimensional adjustment (comprising emotional, occupational, social, family and health adjustment) and general satisfaction. For strengthening the validity of the construct of well-being, the present study adopts both the negative (burnout) as well as the positive (optimism) aspects of employee well-being which have been discussed in the following paragraphs.

### Work engagement and well-being

The question remains if the work engagement leads to employee well-being. Saks (2006) studied organizations with 60% female employees working on a variety of jobs and found that the perceived organizational support predicted both the job and the organization engagement. The job characteristics determined job engagement; and the procedural justice predicted organization engagement. Chung and Angeline (2010) investigated if employee engagement mediates between the job resources and their performance. The results highlighted the need for providing adequate resources to the employees so that they would not only be engaged but will also perform their job well.

Employees with positive self-efficacy and psychological state of minds are energized, involved and engaged with their work. On the contrary, when employees burnout, they manifest signs of exhaustion, lower energy and become more cynical, less involved, and less productive at work (Maslach and Leiter, 1997; Wong and Tay, 2010). Sharma Radha (2005, p. 7) posits “Executive burnout is marked by persistent feelings of inadequacy, ambiguity, dissatisfaction and powerlessness accompanied by behavioral manifestations of apathy and indifference; and physical and emotional exhaustion.”

### Optimism and well-being

The construct of optimism is receiving increasing attention by the positive psychologists and generalists in recent years. Optimism is sometimes treated as a stable personality trait (Scheier and Carver, 1992). Seligman (1975) introduced the concept of learned helplessness which implies that when unpleasant situations become inescapable, employees learn to be helpless and accept the stressors in the environment (Mineka and Hendersen, 1985). He later extended it to the theory of learned optimism (Seligman, 1991/1998). He used attributional approach to explain people's behavior; that pessimists and optimists adopt on habitual basis. He posits that the pessimists hold themselves responsible for failure most of the time and undermine their efforts; whereas the optimists attribute their failures to external factors, which they consider as temporary setbacks due to some situational factors. Gruber-Baldini et al. (2009) in their study assessed the role of optimism/pessimism and locus of control on a sample of patients of Parkinson disease found that greater internal locus of control was associated with less disability and higher optimism was associated with better mental health and quality of life. Luthans (2011, p. 212) considers optimism as a cognitive characteristic with generalized positive outcome expectancy; main stream psychology treats it as an individual difference focusing on cognitively determined expectations. Carver and Scheier (2002, p. 231, Scheier and Carver, 1987) posit that the optimists expect good things to happen to them whereas the pessimists expect the reverse. Optimism has been considered as an indicator of well-being (Scheier and Carver, 1985; Peterson and Bossio, 2002; Scheier et al., 2002). The above studies have used optimism as an independent variable and an indicator of well-being; the present research focuses on the role of perceived gender equity on optimism as a dependent variable and an indicator of positive well-being.

The foregoing discussion yields work engagement to be an under researched area which is likely to play a significant role in the employee well-being, hence it has been selected as a study variable. Thus, based on the knowledge gap identified through the literature review, the following hypotheses have been evolved.

*Hypothesis 1*: There is a positive relationship between the perceived gender equity and work engagement (vigor, dedication and absorption).
*Hypothesis 2*: There is a positive relationship between the locus of control and work engagement.

Bishop (2002) posited that the women's mental well- being is adversely affected by the gender based role and role expectations, responsibility and power relations. According to Madden (2008) women report lower levels of mental well-being across nations. Australian Bureau of Statistics^3^^,^^4^. conducted a survey of women in 2004-05 and found that women reported higher levels of distress than men, and were likely to suffer from long term mental and behavioral problems. Based on the foregoing, the following hypotheses have been formulated.

*Hypothesis 3*: There is a negative relationship between the perceived gender equity and the executive burnout (negative well-being).
*Hypothesis 4*: The work engagement plays a mediating role between the perceived gender equity and internal locus of control (independent variables), and the executive burnout (dependent variable).
*Hypothesis 4a:* The work engagement plays a mediating role between the perceived gender equity and optimism (positive well-being).
*Hypothesis 4b:* The work engagement plays a mediating role between the perceived gender equity and general satisfaction with life and work (positive well-being).
*Hypothesis 5*: There is a positive relationship between the perceived gender equity and employee well-being (optimism).
*Hypothesis 6*: There is a positive relationship between the perceived gender equity, and general satisfaction with life and work (positive well-being).

## Methods

### Samples and procedures

The sample for the study was drawn by stratified random sampling comprising both male and female managers from public and private sectors representing manufacturing and service industry in India. The first researcher approached the HR department of automobile and oil refinery firms for manufacturing and bank/insurance and power transmission for service industry with national presence and having their offices in national capital region of Delhi, India. These were homogeneous groups representing manufacturing and service industry in India. Having obtained the list of managers from the firm, the respondents were selected randomly. They were invited to a common place at a pre-scheduled time. Gender equity being a sensitive subject for employees, a personal survey method was adopted for data collection which, though time consuming and expensive, had many advantages. The first author established rapport with the respondents in a group setting and explained the purpose and importance of the study and sought their informed consent. The present study is a social science research conducted at an academic institute and is not a medical research for which ethics approval is required. The data was collected in face to face situation and the respondents were requested to give the genuine answers and not the ideal ones. The average time taken by the respondents was about 20 min. The advantage of this method was high consistency in the process of data collection as all the respondents received the same introductory instructions; filled the same structured measures, in the same environment with no interference from their organizations. They could clarify their doubts, if any, and were given the option not to reveal their identity, if they so desired. The response rate was 93.22%, much higher than it would have been in a mailed survey. Though they were requested to respond to all the questions, 27 respondents left many questions blank. Consequently, these 27 incomplete questionnaires had to be discarded. Of the 373 participants 191 (51.21%) belonged to the public sector, while 182 (48.79%) to the private sector. 141 (37.8%) belonged to manufacturing firms, while 232 (62.2%) belonged to service organizations. 271 (72.7%) of the survey participants were males, while 102 (27.30%) were females (female percentage is low at managerial level in the Indian Industry). The average age of the participants was 37.6 years. The average work experience was 12.2 years.

### Measures

The study used standardized measures for data collection on the study variables. Besides the indigenous measures, adapted form of other measures was used. All the measures were tested for their validity and reliability on the study sample and their values have been reported in Table 1.

- ***Perceived Gender Equity:*** Scale by the researcher (Sharma Radha, 2013). The questionnaire was developed through literature review, consultation with subject matter experts and HR professionals (Clark and Watson, 1995; Dawis, 1987). It went through pilot testing (Costello and Osborne, 2005) and two more rounds of data collection on samples of 48, 190, and 353 respectively on male and female managerial staff from public and private sectors representing manufacturing and service industry in India. The initial scale had 77 items including some reverse items to take care of social desirability (DeCoster, 2005). Data were collected on 6 response categories ranging from 1 (not at all true) to 6 (always true), a high score indicating high perceived gender equity. The sample adequacy KMO (Kaiser-Meyer-Olkin) measure for the sample was 0.915 which was statistically significant. For *construct validity*, i.e., the extent to which the scale measures the psychological construct, three rounds of exploratory factor analyses were carried using SPSS package. On the basis of the third round of exploratory factor analysis 4 factors were extracted; using principal component method with Varimax rotation. This resulted in total 63 items; out of which 31 items were selected for further analysis on the basis of high factor loadings (above 0.60) and scale-if-item-deleted analysis. These 31 items were then subjected to confirmatory factor analysis (CFA) (Schreiber et al., 2006). The CFA resulted in a measurement model with a total of 29 items, showing good fit with comparative fit index (CFI) = 0.957, incremental fit index (IFI) = 0.958, Tucker and Lewis Index (TLI) = 0.950, RMSEA = 0.044, RMR = 0.094, normed chi-square ≤ 1.678 (chi square = 580.429, *df* = 346, *p* ≤ 0.01. The standardized factor loadings were greater than 0.6 and all were significant at *p* ≤ 0.01 (Aiken and Marnat, 2009). Moreover, the co-variances between the factors were less than 0.80. This established the discriminant and convergent validity for the measurement model of the perceived gender equity showed that the purified measure of this construct was acceptable psychometrically.
  The final Perceived Gender Equity scale comprises of 29 items with three dimensions labeled as “Equity perception through organizational policies”; “Equity perception through organizational practices” and “Equity perception through organizational environment.” Some of the sample items for Equity perception through organizational policies are (Organizational policy reflects commitment toward fair treatment of both male and female employees), (Both men and women in the organization are provided adequate developmental opportunities to further their career goals). For equity perception through organizational practices items are (Women in leadership positions are scrutinized closely by their male colleagues), (For senior leadership roles male employees are preferred). For equity perception through organizational environment a sample item is (The organization provides a conducive work-environment so that both male and female employees can contribute toward organizational goals). The Cronbach alpha (α) value for the scale is 0.933 and comparative fit index (CFI) = 0.957, incremental fit index (IFI) = 0.958, Tucker and Lewis Index (TLI) = 0.950.
- ***Work and Well-being survey***: (UWES) by Schaufeli and Bakker (2003; Schaufeli et al., 2006) consists of 17 statements with 6 response categories ranging from 0 (never) to 6 (always/every day). It measures the work engagement on three sub scales: *Vigor, Dedication and Absorption*. The scale has been tested in 10 countries by the test developers. Its Cronbach alpha on the study sample is 0.937.
- ***Locus of Control***: LOC scale by Rotter (1966) is a measure of internal and external LOC. It contains 10 pairs of statements and the respondent has to choose one statement between the pair which matches with his thinking. High scores on the scale indicate internal locus of control. Its Cronbach alpha on the study sample is 0.668 and model fit indices NFI = 0.985, IFI = 0.991, TLI = 0.985, and CFI = 0.991.
- ***Executive Burnout Scale:*** An Executive Burnout Scale developed by Sharma Radha (2005, 2007) contains 28 items and measures five dimensions of executive burnout viz., *ambiguity, inadequacy, dissatisfaction and powerlessness, depersonalization and physical and emotional exhaustion*. Its Cronbach's alpha on Indian executive sample was 0.910 and its concurrent validity with Maslach Burnout Scale was 0.350 (*p* < 0.01) (Sharma Radha, 2005). Its Cronbach alpha on the study sample is 0.963 and model fit indices are NFI = 0.996, IFI = 0.997, TLI = 0.992, and CFI = 0.997. High scores on the scale indicate executive burnout.
- ***Life Orientation Test***: Optimism Life Orientation Test (LOT-R) has been developed by Scheier et al. (1994). The correlation between the revised and the original scale is 0.95. It contains 10 items and five response categories. Its Cronbach alpha on the study sample is 0.500 and its model fit indices are NFI = 0.996, IFI = 0.996, TLI = 0.997, and CFI = 0.999. High scores on this scale indicate high optimism and a score of zero indicates extreme pessimism.
- ***General Satisfaction with Life and Work Scale:*** A Likert type scale developed by Sharma Radha (2011) in the Indian context was used to measure general satisfaction with life and work of the managers. It consists of 6 items and is a one factor scale. Its Cronbach alpha on the study sample is 0.884 and its model fit indices are NFI = 0.998, IFI = 0.999, TLI = 0.997, and CFI = 0.999. High scores on this scale indicate satisfaction with life and work.

**Table 1:** **Measures used for the study**.

**Scale**	**Measure developed by/Source**	**Factors**	**Original items**	**Total no. of items selected**	**Cronbach alpha on the study sample**	**CFA: Model fit indices**
Work engagement	Schaufeli and Bakker, 2003	3	17	17	0.937	3 factor model confirmed by factor analysis
General satisfaction with life and work	Sharma Radha, 2011	1	8	6	0.884	NFI = 0.998 IFI = 0.999 TLI = 0.997 CFI = 0.999
Executive burnout	Sharma Radha, 2005, 2007	5	28	28	0.963	NFI = 0.996 IFI = 0.997 TLI = 0.992 CFI = 0.997
Internal locus of control	Adapted from Rotter, 1966	2	20	10	0.668	NFI = 0.985 IFI = 0.991 TLI = 0.985 CFI = 0.991
Optimism	Adapted from Scheier et al., 1994	1	6	4	0.500	NFI = 0.996 IFI = 0.996 TLI = 0.997 CFI = 0.999
Perceived gender equity scale	Sharma Radha, 2013	3	29	29	0.933	IFI = 0.958 TLI = 0.950 CFI = 0.957

*All statistics based on analysis of the authors*.

Cronbach alpha and CFA were determined for all the adapted standardized measures on the study sample (Table 1) which indicate reliability and validity of the measures.

### Control variables

Based on earlier studies (Avolio et al., 2004) age, education and managerial level were treated as control variables for all the respondents in the analysis.

## Results

### Basic statistics and correlations

Means, standard deviations and correlations among variables are reported in Table 2. These indicate hypothesized positive relationship between the perceived gender equity and work engagement; locus of control and work engagement; perceived gender equity and general satisfaction with work and life; and negative relationship between perceived gender equity and executive burnout, all statistically significant (*p* ≤ 0.01). A significant negative relationship (*p* ≤ 0.01) has been observed between perceived gender equity and optimism; a possible explanation could be that equity is not a superficial phenomenon; there are umpteen number of cases of covert discrimination at the workplace in India, therefore, the respondents might not have been optimistic in their outlook. The work engagement yields significant positive relationship with optimism, general satisfaction with life and work and negative correlation with executive burnout.

**Table 2:** **Means, standard deviations, alpha reliabilities and inter-correlations**.

**Variables**	**Mean**	***SD***	**1**	**2**	**3**	**4**	**5**
1. Perceived gender equity (PGE)	126.74	23.72					
2. Internal locus of control (ILOC)	7.06	2.01	0.20^**^				
3. Work engagement (WE)	71.17	17.73	0.32^**^	0.42^**^			
4. Optimism (OPT)	9.25	2.91	−0.18^**^	−0.38^**^	0.29^**^		
5. General satisfaction with life and work (GSLW)	22.87	3.82	0.23^**^	0.22^**^	0.37^**^	−0.19^**^	
6. Executive burnout (BUNT)	59.16	27.94	−0.29^**^	−0.47^**^	−0.56^**^	0.39^**^	−0.40^**^

** *p ≤ 0.01*.

*Analysis by the authors*.

During the model testing phase, data analysis was carried out for a total of 373 respondents using the statistical package for social science version 10.0 (SPSS 10.0) and the analysis of moments structure (AMOS) version 4.0 The data analysis process entailed two phases: (1) scale analysis and measurement model analysis for each measurement instrument through CFA to ensure its reliability and validity (Podsakoff et al., 2003) and (2) path model analysis using structural equation modeling (SEM) and partial least squares techniques encompassing maximum likelihood estimation (MLE).

### Path analysis

Fit tests were used to test the path model that was built on the basis of hypotheses generated through the literature review. The mediation by the work engagement was tested through SEM and by using the procedures recommended by Baron and Kenny (1986). As suggested by Arbuckle and Wothke (1999) and MacCallum and Austin (2000), root mean square error of approximation (RMSEA), incremental fit index (IFI), Tucker Lewis index (TLI), comparative fit index (CFI) and normed chi-square were used as the fitness criteria to test latent variable model fit and contrast the direct effects path model with the mediating effects path model for mediation analysis using nested models (Long, 1983).

To take care of common method bias as all the respondents of the present study had responded to all the measures using a single survey instrument at a given time, the researchers conducted the Harman's single factor test. A factor analysis was carried out including all the variable items used in the study to check whether a disproportionately large variance was being claimed by a single factor. Analysis of results confirmed absence of a single factor accounting for the majority of the variance and hence, absence of common method bias. In order to minimize the common method variance (Podsakoff et al., 2003) the dependent variable of the employee well-being has been conceptualized covering both positive and negative aspects. For the positive aspect “optimism” and “general satisfaction with life and work” have been chosen and for the negative aspect “executive burnout” has been used. These have been assessed using standardized measures.

An examination of the relationships among the constructs was carried out on the basis of standardized regression estimates (β) for the accepted path model, depicted in Table 3. As suggested by Byrne (2001), the level of significance was estimated on the basis of critical ratio (CR) values corresponding to the regression estimates. CR values equal to or greater than 2.58 indicated a significance level of 99% and, CR values less than 2.58 but greater than or equal to 1.96 indicated a significance level of 95%.

**Table 3:** **Hypotheses testing through regression estimates**.

**Path from**	**Path to**	**Unstandardized coefficients**		**Standardized coefficients**			**Remarks**
		**β**	**Standard error**	**β**	**CR**	***p***	
Perceived gender equity	Work engagement	0.646	0.144	0.261^**^	4.480	*p* ≤ 0.01	H1 accepted
Internal locus of control	Work engagement	14.897	2.284	0.428^**^	6.524	*p* ≤ 0.01	H2 accepted
Perceived gender equity	Executive burnout	−0.064	0.045	−0.075^***^	−1.442	*p* ≤ 0.10	H3 accepted
Work engagement	Executive burnout	−0.132	0.021	−0.380^**^	−6.254	*p* ≤ 0.01	H4 accepted
Work engagement	Optimism	−0.041	0.007	−0.491^**^	−5.513	*p* ≤ 0.01	H4a accepted
Work engagement	General Satisfaction with life and work	0.033	0.005	0.414^**^	6.771	*p* ≤ 0.01	H4b accepted
Perceived gender equity	Optimism	−0.026	0.015	−0.124^***^	**-**1.680	*p* ≤ 0.10	H5 rejected
Perceived gender equity	General Satisfaction with life and work	0.022	0.012	0.115^***^	1.859	*p* ≤ 0.10	H6 accepted

** p ≤ 0.01;

*** *p ≤ 0.10*.

*Analysis by the authors*.

The standard regression coefficient values are as follows: the perceived gender equity is related to the work engagement (β = 0.261, *p* ≤ 0.01); the internal locus of control and the work engagement (β = 0.428, *p* ≤ 0.01); the perceived gender equity and the executive burnout (β = −0.075, *p* ≤ 0.10); the perceived gender equity and the optimism (β = −0.124, *p* ≤ 0.10) and the perceived gender equity and the general satisfaction with life and work (β = 0.115, *p* ≤ 0.10). The results support the Hypotheses 1, 2, 3, 5, and 6 respectively.

**Figure 1:**
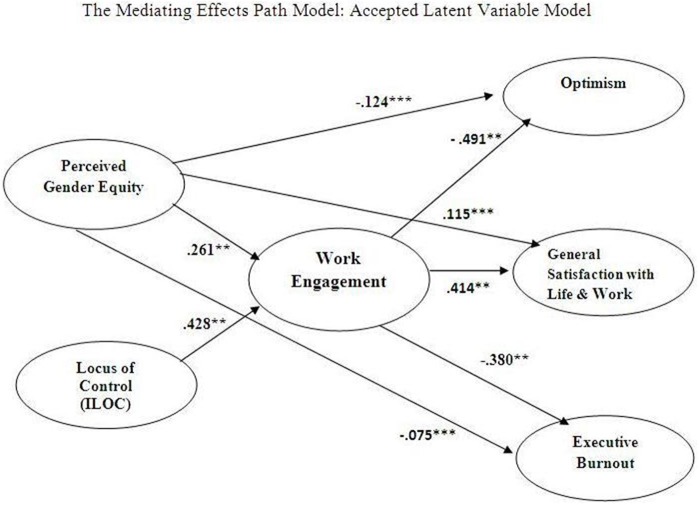
**Model for perceived gender equity and locus of control and mediation of work engagement in employee well-being**.

As suggested by the theory and stated in Hypotheses 4, 4a, and 4b, the Mediating Effects model (vide Figure 1) posited that the relationship between the perceived gender equity and locus of control (independent variables) and the executive burnout (dependent variable) was mediated by the work engagement. It also implied that the relationship between the perceived gender equity and optimism as well as the relationship between the perceived gender equity and general satisfaction was also mediated by the work engagement. Whereas, the Direct Effects model conjectured a direct relationship between the perceived gender equity, locus of control as exogenous variables and the optimism, general satisfaction and executive burnout as endogenous variables.

The causal model comparison analysis based on the fitness criteria, as depicted in Table 4, suggested that the Mediating Effects model be accepted. The chi square of the Mediating Effects model was 831.19 (*df* = 424) and the chi square of the Direct Effects model was 739.30 (*df* = 345). The chi square difference between the two models was significant (i.e., 91.89). These results support our Hypotheses 4, 4a, and 4b.

**Table 4:** **Nested models analysis of results for mediating effects model and direct effects model**.

**Mediating effects model**			**Direct effects model**		
**Path from**	**Path to**	**Standardized regression coefficient β**	**Path from**	**Path to**	**Standardized regression coefficient β**
Perceived gender equity	Work engagement	0.261^**^	Perceived gender equity	Optimism	−0.399^**^
Internal locus of control	Work engagement	0.478^**^	Perceived gender equity	General satisfaction	0.334^**^
Work engagement	Optimism	−0.491^**^	Perceived gender equity	Executive burnout	−0.229^**^
Work engagement	General satisfaction	0.414^**^	Internal locus of control	Executive burnout	−0.517^**^
Work engagement	Executive burnout	−0.380^**^			
Perceived gender equity	Optimism	−0.124^***^			
Perceived gender equity	General satisfaction	0.115^***^			
Perceived gender equity	Executive burnout	−0.075^***^			
Internal locus of control	Executive burnout	−0.362^**^			
RMSEA	0.051		RMSEA	0.055	
IFI	0.986		IFI	0.984	
TLI	0.984		TLI	0.981	
CFI	0.986		CFI	0.984	
χ^2^	831.198		χ^2^	739.301	
df	424		df	345	
χ^2^/df	1.96		χ^2^/df	2.143	

** p ≤ 0.01;

*** *p ≤ 0.10*.

*Analysis by the authors*.

## Discussion and implications

The acceptance of Hypothesis 1 (H1) indicates that the perceived gender equity is positively related to the work engagement (Table 3). Theoretically, it extends the work engagement model proposed by Schaufeli et al. (2006) with the findings of the present study that the perception of the workplace gender equity is a significant predictor of engagement at work. Besides, the perception of gender equity results also confirm the role played by employees' locus of control in this context. Halloran et al. (1999) posited that people high on the internal LOC believe that their behavior is directly linked with the outcome because they have control over their environment. Hypothesis 2 states that there is positive relationship between the internal locus of control and the work engagement (vigor, dedication and absorption). The support for this hypothesis has led us to theorize that individuals with high internal locus of control often try taking charge of the situation themselves and engage themselves in work and thereby derive satisfaction; this further augments the employee well-being. Employees with the internal locus of control are less prone to burnout as they determine their course of action and work accordingly. These findings also contribute significantly to the future gender based research besides having policy implications for the organizations. These findings find support in the study by Spector (1982) (who posited that people with internal LOC have more positive self-evaluation, better performance, are more satisfied with their job and have better coping skills in stressful situations) and tend not to burnout (Glassgow, 1986).

The acceptance of hypothesis 3, that there is a negative relationship between perceived gender equity and executive burnout, empirically supports the notion that perception of gender equity at the workplace is expected to enhance feelings of adequacy, empowerment, physical and emotional well-being. When the employees perceive equity at the workplace, they are likely to perform optimally and feel less stressed.

Hypothesis 4 is that the work engagement mediates the relationship between the perceived gender equity and ILOC (independent variables), and executive burnout (dependent variable). Hypothesis 4a posits that the work engagement mediates the relationship between the perceived gender equity and optimism. Hypothesis 4b is that the work engagement mediates the relationship between the perceived gender equity and general satisfaction with life and work. The support provided for these two hypotheses (Table 4) confirms the mediating role of the work engagement in the relationship between the perceived gender equity, internal locus of control (independent variables) and the employee well-being. This indicates that perception of gender equity at the workplace along with employees' internal locus of control can result in enhanced employee engagement, which in turn can lead to employees' general satisfaction with life and work and reduce incidence of executive burnout.

Hypothesis 5 that the perceived gender equity is positively related with optimism was not supported. This made us explore that why perception of gender equity at the workplace bears a negative relationship with optimism in the Indian context. Since, the majority of our respondents comprised of males, we decided to carry out separate correlation analysis for males and females in our sample and found that both the male (*n* = 271) and the female (*n* = 102) samples have negative correlations between the perceived gender equity and optimism. While the correlation for the males −0.175 is significant at ≤ 0.01 level; it is only −0.161 and non-significant for the females. This leads us to assume that while the male employees considered gender equity to be gender bias at the workplace, the female employees have reconciled to the gender discrimination over the years. Hence, their optimism is not governed by their perceived gender (in) equity at their respective workplaces. This can be explained by the concept of learned helplessness (Seligman, 1975; Mineka and Hendersen, 1985) which explains that when stressful or unpleasant situations become inescapable, employees learn to be helpless and accept these, even when change for the better is possible. There is also a possibility of cultural difference playing a role in this finding as the Chronbach alpha for the optimism scale (developed abroad) on the study sample was 0.5. but observing the social reality and significantly high negative correlation for the male employees, the possibility of women employees reconciling to inequity is high. Therefore, there is an urgent need for altering the gender equity related perceptions amongst both the male and the female employees in the Indian context. Having gender equity policies is not sufficient in itself; it requires a change of mindset by various interventions such as more women in the corporate board, change in the work environment and workplaces practices to mitigate the gender discrimination at workplaces in the truest sense.

Except a study by Fiksenbaum et al. (2010), who investigated the relationship between the two virtues of optimism and proactive behavior and psychological well-being amongst working women in the Turkish banking sector, one scarcely comes across a research linking the well-being and gender at work. Hence, this is possibly the first empirical study to report that perceived gender equity at the workplace has diverse implications for the optimism dimension of well-being amongst the male and the female employees. Further, acceptance of hypothesis 6 that there is a positive relationship between the perceived gender equity and general satisfaction with life and work confirms positive correlation between the perceived gender equity and employee well-being. We conclude that the perceived gender equity impacts various dimensions of the employee well-being (viz. optimism, general satisfaction with life and work, and executive burnout) differently due to not only to the organizational policies but also due to the organizational practices and the nature of (in) equitable work environment.

Thus, from a practitioner's perspective, we suggest that managers need to ensure the perceived gender equity through organizational policies of recruitment, training and development, compensation and career progression. Attention needs to be paid to the workplace practices and environment so that employees are treated with professional respect and dignity which will enhance work engagement and employee well-being. We also recommend that HR managers take into consideration their national and cultural contexts for implementing the gender equity policies, practices and gender diversity initiatives. While planning their human resource development and employee engagement initiatives, efforts need to focus on enabling the employees (specifically women) to optimally grow their potential and to enjoy their work. The need for gender equity initiatives and incorporation of gender sensitive inputs in the education programmes cannot be over emphasized to pass on the values about gender equity and gender equality to the future generations (Leskaj and Guga, 2009). In this regard International Centre for Research on Women, in collaboration with local institutions, has developed and implemented a curriculum to engage young girls and boys, age between 12 and 14 years in various countries (http://www.icrw.org/ accessed on 14.6.15). Similar initiatives can be adopted to promote gender equity in the geographies where gender discrimination is high. Audio-visual and social media can play an effective role in sensitizing the young generation about gender equity so that they enter the world of work with a progressive mindset. Building organization culture encompassing gender equity would go a long way in creating a humanistic society therefore; firms need to promote the gender equity at the workplace by formulation of apposite policies, adoption of practices, and creation of an equitable environment through an appropriate perceptual and attitudinal change.

## Limitations and future directions

The study involves limited variables viz., the perceived gender equity, locus of control, work engagement, optimism, satisfaction with life and work and executive burnout. Future studies could consider other variables like gender equality as independent variable and determine its effect on the work engagement and employee well-being. The optimism scale yielded Chronbach alpha value of 0.5; this could be considered a limitation of the study; the future studies could develop an indigenous scale. The Other methodologies could also be considered, e.g., case study can be adopted to focus on organizational policies and practices to promote gender equity. Longitudinal study of women managers may bring greater insight into the phenomenon of gender inequity and how it affects their work engagement and well-being. Future studies could also focus on the effect of the perceived gender equity on the organizational outcomes. Cross cultural studies would be helpful identifying determinants of well-being of women managers across geographies. Future studies could use large samples or industry specific samples to gain industry specific insights.

## Contributions

The study makes theoretical contribution by providing a definition for gender equity at the workplace which can be used by scholars for future research. The study can be replicated/adapted in other countries which face the problem of gender inequity. Most of the studies on women have been done on work–family conflict, commitment and supervisory support and have ignored equity issues because of the complexity involved in defining the construct of gender equity.

The findings of the study inform the extant literature how the perceived gender equity and internal locus of control affect work engagement, an important variable in organizational psychology and human resource management. The work engagement would not only contribute to organizational productivity but will also enhance satisfaction with life and work and the well-being of employees, thus creating a positive workplace.

The present study contributes to the extant literature on the employee well-being which is an emerging area of study in positive Psychology. The study has adopted three measures for employee well-being- two for assessing positive aspects (optimism, satisfaction with life and work) and one for measuring negative aspect (executive burnout) thus expanding the conceptualization of the construct of well-being. It not only provides another perspective to the concept of employee well-being and but also provides convergent and divergent validity of the construct of employee well-being.

Well-being is garnering attention of both the scholars and practitioners due to stressful work environment, a concomitant of volatile economic environment, which is impacting employees in general and has made women employees more vulnerable. By identifying the determinants of employee well-being at the workplace the study provides empirical data for taking policy decisions for promoting gender equity at the workplace and thereby enhancing well-being of employees. Self-interest theory (Lind and Tyler, 1988; Sears and Funk, 1991) yields that employee behavior is governed by salient personal gains to maximize the utility of their actions. Organizations take initiatives for enhancing productivity of the employees in general and often ignore initiatives for the women employees who could be a great resource, if treated with equity. Butts et al. (2010) report that work-family programmes provide value to firms in terms of positive press coverage and popularity. Thus, the findings make a case for female talent management which will indirectly help the organizations boost their image and reputation. The research findings could lead to development of gender specific theories of work engagement and employee well-being.

## Conflict of interest statement

The authors declare that the research was conducted in the absence of any commercial or financial relationships that could be construed as a potential conflict of interest.

## Footnotes

^1^22 official languages.

^2^India's gender ratio figures are 943 females to 1000 males according to Census of India, 2011. (http://www.iwpr.org/initiatives/pay-equity-and-discrimination May1, 2015.

^3^http://www.abs.gov.au/AUSSTATS/abs@.nsf/ProductsbyCatalogue/3AB354FFA0B0A31FCA256F2A007E5075?OpenDocument.

^4^http://www.thehindubusinessline.com/2010/03/09/stories/2010030952811800/.
